# Histological findings in post-extraction sockets after alveolar ridge preservation with leukocyte- and platelet-rich fibrin: a randomized clinical trial

**DOI:** 10.1590/1678-7765-2026-0283

**Published:** 2026-09-18

**Authors:** Maria Pavli, Maria Georgaki, Nadia Theologie-Lygidakis, Nikolaos Nikitakis, Christos Perisanidis

**Affiliations:** 1 National and Kapodistrian University of Athens School of Dentistry Department of Oral and Maxillofacial Surgery Athens Greece National and Kapodistrian University of Athens, School of Dentistry, Department of Oral and Maxillofacial Surgery, Athens, Greece.; 2 National and Kapodistrian University of Athens School of Dentistry Department of Oral Medicine and Pathology and Hospital Dentistry Athens Greece National and Kapodistrian University of Athens, School of Dentistry, Department of Oral Medicine and Pathology and Hospital Dentistry, Athens, Greece.

**Keywords:** Leukocyte platelet rich fibrin, Tooth socket, Alveolar ridge, Dental implants, Histology

## Abstract

**Introduction:**

Leukocyte- and platelet-rich fibrin (L-PRF) has gained considerable attention due to its regenerative potential and biocompatibility in alveolar ridge preservation (ARP).

**Objective:**

To evaluate the histological findings in post-extraction sockets after ARP with L-PRF (experimental group) and compare them with those obtained using allograft plus a collagen membrane (control group). Histological findings may contribute to the evaluation of bone quality and the rate of bone remodelling.

**Methodology:**

Twenty-six sites in 26 patients requiring extraction of a single-rooted maxillary or mandibular tooth were included in this study. Treatment options were randomly assigned. Post-extraction sockets were filled with either an L-PRF matrix or an allograft covered with a collagen membrane. Dental implant placement was performed subsequently. After 12 weeks, bone biopsies were obtained from the sites at the time of implant placement. Histological evaluation of the bone specimens assessed the area of new bone formation and the presence of osteoblasts, osteoclasts, osteolytic lacunae, osteocytes, and blood vessels.

**Results:**

Histological evaluation showed that: a) the proportion of cases with >50% new bone formation was higher in the experimental group compared to the control group (53.8% [7/13] versus 30.8% [4/13]) (p=0.033) b) the presence of osteoblasts was higher (p=0.066) in the experimental group (100% versus 76.9%); c) the presence of osteoclasts was higher (p=0.004) in the experimental group (92.3% versus 38.5%); d) the presence of osteolytic lacunae was higher (p=0.063) in the experimental group (92.3% versus 61.5%); e) the presence of osteocytes was higher (p=0.03) in the experimental group (100% versus 69.2%); and f) the presence of blood vessels was higher (p=0.016) in the experimental group (84.6% versus 38.5%). The presence of osteoblasts and osteolytic lacunae were higher but were not statistically significant.

**Conclusions:**

Within the limitations of this study, ARP with L-PRF may result in accelerated healing, improved bone quality, and a higher rate of bone remodelling. These results reflect the exploratory nature of this study and support the need of additional research, preferably with a larger sample size.

## Introduction

Tooth extraction causes bundle bone loss and bone remodelling, resulting in a reduction in alveolar ridge dimensions.^1^ This process is more pronounced in the anterior region of the jaw and particularly following a traumatic extraction involving the buccal cortical plate compared to the palatal/lingual plate.^2^ These changes may compromise implant placement and prosthetic rehabilitation.^2^ Therefore, alveolar ridge preservation (ARP) following tooth extraction is a crucial prerequisite for achieving optimal functional and aesthetic outcomes in implant therapy. ARP was introduced to reduce, although not completely prevent, the physiological bone resorption that occurs immediately after tooth extraction by placing a grafting material into the extraction socket.

Various techniques and biomaterials have been used for ARP and guided bone regeneration (GBR), including filling the post-extraction socket with bone substitutes, sealing the socket opening with a barrier material, or a combination of both.^3^ A variety of bone grafting materials from different sources have been used, including human-derived, animal-derived, and synthetic materials. The most commonly used bone graft substitutes are allografts and xenografts. Demineralized allografts, including freeze-dried bone allograft (FDBA) and demineralized freeze-dried bone allograft (DFDBA), have osteoinductive properties and a fast resorption rate, thereby enhancing bone regeneration while maintaining space for new bone formation.^4,5^ Xenografts have osteoconductive properties and a slow resorption rate, which may contribute to maintaining bone volume.^6^ Collagen membranes prevent epithelial migration, stabilize the graft, and facilitate guided bone regeneration according to Melcher’s GBR principles.^7^ The combination of an allograft and a collagen membrane is considered a widely accepted and highly predictable technique for ARP.^8,9,10^

Several randomized clinical and histological studies have shown that allograft combined with a collagen membrane reduces ridge resorption compared to natural healing. Iasella, et al.^8^ (2003), in a randomized control trial (RCT) evaluating ARP with FDBA and a collagen membrane, concluded that there were significant increases in both horizontal and vertical dimensions compared to natural healing. In the clinical study by Barone, et al.^9^(2008), the combination of a xenograft and a collagen membrane yielded similar results, with a decreased resorption rate and improved implant site morphology.^9^ Avila-Ortiz, et al.^10^ (2014), in a systematic review, concluded that ARP with allograft materials has a significant treatment effect in buccolingual width (1.89mm), midlingual height (1.18mm) and midbuccal height (2.07mm) compared to natural healing. Furthermore, histological evaluation of sites preserved with an allograft and a collagen membrane demonstrated a higher proportion of vital bone and favorable remodelling characteristics.^4,11^ In conclusion, randomized clinical trials, histological studies, and systematic reviews support the use of an allograft combined with a collagen membrane as a predictable method for ARP of post-extraction sites.

Autologous platelet concentrates have attracted significant interest due to their regenerative potential and biocompatibility. Platelet-rich fibrin (PRF) and leukocyte- and platelet-rich fibrin (L-PRF), introduced by Choukroun, et al.^12^ (2001), represent second-generation platelet concentrates produced using a simplified, anticoagulant-free centrifugation protocol to obtain a fibrin matrix enriched with platelets, leukocytes, and a high concentration of growth factors. Advanced-platelet rich fibrin (A-PRF) was subsequently introduced by Ghanaati, et al.^13^ (2014) as an advancement of the original PRF. A-PRF uses a different preparation protocol and results in a more porous fibrin matrix that releases growth factors over a longer period.^13^ The autologous and cost-effective nature of these materials, combined with their capacity to accelerate wound healing and enhance osseointegration, supports their growing use in regenerative and implant dentistry.

Grafting extraction sockets with active infection (e.g., severe periodontal lesions or periapical pathology) was traditionally contraindicated due to high graft failure rates. PRF variants have challenged this concept because of their high leukocyte concentration, which may provide sustained immunological protection.^14^ Chen, et al.^14^ (2019), documented the successful preservation of sockets with severe buccal wall defects and localized infection. The study highlighted the capability of PRF to suppress inflammatory responses and promote tissue repair in contaminated environments, provided that infected tissues are removed.

The combination of PRF with particulate bone grafts, widely known as “sticky bone,” has gained considerable attention as a biologically enhanced regenerative approach in ARP, GBR, and implant dentistry. The continued release of these bioactive molecules enhances angiogenesis, mesenchymal stem cell recruitment, osteoblast differentiation, and extracellular matrix formation while modulating the inflammatory response, thereby creating a favorable microenvironment for bone regeneration.^13,15^ Additional evidence supporting the biological advantages of sticky bone was provided by Zheng, et al.^16^ (2026), who investigated A-PRF combined with xenogeneic bone substitutes in periodontally compromised extraction sockets. Their radiographic analysis demonstrated that ARP using sticky bone in combination with an A-PRF membrane was associated with more favorable dimensional and volumetric outcomes than natural healing in periodontally compromised extraction sockets.^16^ Sareen, et al.^17^(2024), in a systematic review, further described the biological rationale and clinical applications of sticky bone, in which liquid PRF acts as a natural autologous fibrin binder, forming a three-dimensional fibrin scaffold that stabilizes particulate grafts while simultaneously delivering regenerative cells and signalling molecules directly to the surgical site. Additionally, Yang, et al.^18^ (2025) emphasized that liquid PRF promotes vascular ingrowth, accelerates soft tissue closure, and facilitates bone remodelling through continuous release of growth factors over several days following implantation.

Collectively, the available evidence indicates that sticky bone represents a regenerative strategy in which the mechanical advantages of graft stabilization are combined with the biological effects of autologous platelet concentrates.

The synergistic interaction between autogenous dentin matrix and PRF in ARP has also been studied. Autogenous dentin represents an ideal grafting material because it shares remarkable compositional and structural similarities with alveolar bone, consisting of approximately 70% hydroxyapatite, 20% organic matrix—predominantly type I collagen—and 10% water. Following demineralization, dentin exposes a wide range of bioactive molecules, including bone morphogenetic proteins (BMP-2, BMP-4, and BMP-7), transforming growth factor-β (TGF-β), insulin-like growth factors (IGFs), fibroblast growth factors (FGFs), and other non-collagenous proteins that contribute to osteoinduction and tissue regeneration.^19,20^ When combined with liquid PRF, these biological properties are further enhanced with the continuous release of autologous growth factors and the formation of a favorable microenvironment for angiogenesis, mesenchymal stem cell recruitment, osteoblast differentiation, extracellular matrix deposition, and coordinated bone remodelling. Similarly, Amer, et al.^21^ (2025), in a randomized controlled clinical trial, showed that the combination of injectable PRF and autogenous demineralized dentin significantly enhanced ARP by improving bone quality and maintaining ridge dimensions compared with dentin grafting alone. These outcomes support the hypothesis that PRF not only improves the biological performance of dentin grafts but may also accelerate their integration with the host bone.

Moreover, a clinical and histomorphometric trial investigated autologous tooth-derived granules combined with i-PRF.^22^ The study revealed excellent osteointegration, with newly formed trabecular bone directly surrounding the dentin granules without intervening fibrous tissue.^22^ Based on these findings, the sticky tooth concept appears to represent a biologically enhanced regenerative strategy for ARP.

Clinical studies have reported enhanced soft tissue closure, reduced postoperative discomfort, and improved bone quality in sockets treated with L-PRF compared to conventional healing.^23,24^ Unlike first-generation platelet concentrates such as platelet-rich plasma (PRP) and PRF, L-PRF promotes a sustained release of cytokines and growth factors, including platelet-derived growth factor (PDGF), transforming growth factor-beta (TGF-β), vascular endothelial growth factor (VEGF), and insulin-like growth factor (IGF), which collectively contribute to angiogenesis, osteogenesis, and accelerated soft tissue healing.^25,26^

The aim of this RCT was to present the histological findings of post-extraction sockets and evaluate the rate of bone remodelling and qualitative bone characteristics in comparable cases in which ARP was performed using either L-PRF or an allograft combined with a collagen membrane. The primary outcome measure was the histological percentage of new bone formation after socket preservation between the two groups. Secondary outcome measures included histomorphometric analysis, as well as the percentage of presence of osteoblasts, osteoclasts, osteolytic lacunae, osteocytes, and blood vessel formation.

The null hypothesis was that there would be no significant difference in bone resorption or the degree of bone remodelling in post-extraction sockets between the L-PRF group and the allograft plus collagen membrane group.

## Methodology

### Study Design

Twenty-six patients (15 females and 11 males) requiring extraction of a single-rooted tooth were included in the study. A total of 26 teeth were extracted, comprising 16 maxillary teeth (four incisors, four canines, and eight first premolars) and 10 mandibular teeth (two incisors, six first premolars, and two canines). Post-extraction sockets were randomly assigned to receive either L-PRF matrix or an allograft combined with a collagen membrane. All treatments were performed at the Clinic of Oral and Maxillofacial Surgery, School of Dentistry, National and Kapodistrian University of Athens, Greece. The study was approved by the Institutional Ethics Committee (415/23.05.2019) and registered in the Clinical Trials.gov database (ClinicalTrials.gov ID: NCT07387913). The study was performed in strict accordance with the World Medical Association Declaration of Helsinki, as revised in 2013 (64^th^ WMA General Assembly, Fortaleza, Brazil) and the CONSORT 2025 statement.

### Sample size calculation

Sample size calculation was performed a priori using G*Power software (version 3.1, Heinrich Heine University, Düsseldorf, Germany). The primary endpoint selected for the power analysis was the percentage of new bone formation assessed by histomorphometric evaluation. A two-sided significance level (α) of 0.05 and a statistical power (1−β) of 80% were adopted in accordance with established recommendations for the design of randomized clinical trials.^27^

In the absence of directly comparable preliminary quantitative data from our study population at the time of study design, a large standardized effect size (d=0.80) was assumed a priori. This assumption was considered clinically appropriate based on previously published randomized controlled clinical trials evaluating alveolar ridge preservation with L-PRF, which consistently demonstrated clinically meaningful improvements in histomorphometric new bone formation compared with spontaneous healing.^24,28^ Due to the limited availability of eligible patients meeting inclusion criteria within the study period, recruitment yielded 13 specimens per group. This corresponds to a post-hoc statistical power of approximately 50% to detect the assumed large effect size (d=0.80), and the primary outcome findings should therefore be interpreted as exploratory rather than confirmatory. We acknowledge that this effect size was not derived from quantitative means and standard deviations extracted from the cited trials, but rather from the qualitative consistency of their reported outcomes. This assumption is discussed further as a study limitation.

### Patient Selection - Inclusion/exclusion criteria

Patients received a detailed explanation of the study and provided written informed consent before enrolment. Patient selection was based on clinical and radiographic evaluation.

The inclusion criteria were: 1) no relevant medical history; 2) adult patients; 3) a single-rooted tooth requiring extraction due to caries, trauma, fracture, endodontic failure, or orthodontic reasons; 4) at least one adjacent tooth at the extraction site; 5) intact socket walls or mild to moderate dehiscence (Type I and II); 6) a buccal plate thickness of at least 1.5 mm; and 7) either a thick or thin gingival phenotype.

The exclusion criteria were: 1) medical conditions or medications that could affect bone healing; 2) periodontal disease affecting the tooth to be extracted; 3) a full-mouth plaque index >30%; 4) clinical and/or radiographic evidence of loss of the buccal bone wall after extraction; and 5) smoking more than 10 cigarettes per day.

### Randomization

The treatment allocation codes (“1” for L-PRF and “2” for allograft + collagen membrane) were concealed in sealed, opaque envelopes and opened by an assistant immediately after tooth extraction to determine the treatment allocation.

### Blinding

Blinding was implemented as follows, in accordance with CONSORT recommendations:

- Participants were informed of their assigned group (experimental or control) only after the sealed envelope was opened following atraumatic tooth extraction.
- The clinician became aware of the assigned group (experimental or control) only after the sealed envelope was opened following atraumatic tooth extraction.
- The oral pathologist who examined the specimens was blinded to group allocation.
- The statistician/data analyst was blinded to group allocation.

### Treatment Protocol

All patients who fulfilled the inclusion criteria underwent clinical and radiographic examination. They also received periodontal and restorative treatment, when required, in addition to detailed oral hygiene instructions, before the surgical procedure.

After administration of local anaesthesia (4% articaine with epinephrine 1:100.000; Septanest, Septodont, France) teeth were extracted atraumatically using luxators (Luxator Periotome, Directa, Germany) without flap elevation. The extraction socket was curetted and irrigated with saline solution. The bony walls were carefully examined with a probe to ensure that each case met the inclusion criteria.

To prepare the L-PRF plugs, four 9 ml red-cap blood collection tubes were obtained from each patient. The tubes were centrifuged at 2700 rpm (400 g) for 12 min using a tabletop centrifuge (L-PRF Intraspin System, Intra-Lock International Inc., Boca Raton, FL, USA). The fibrin clots were carefully separated from the middle portion using a spatula, preserving the lower portion of the clot, which is rich in platelets and leukocytes.^24^ The clots were then compressed for 5 min using the appropriate kit (Intra- Lock International Inc. Boca Raton, FL, USA), to produce L-PRF plugs. In the experimental group, several plugs were placed in the extraction socket to completely fill the socket. In the control group, Phoenix TBF allograft particles (TBF, France) were used to fill the extraction sockets, which were then covered with a type I collagen membrane (Biocollagen, Bioteck, Italy). Subsequently, figure-of-eight sutures were placed using 4-0 polydioxanone sutures (Monosorb, Medipack, Greece) in both groups.

Patients received thorough postoperative instructions and were prescribed ibuprofen 600 mg (Algofren, Intermed, Greece) two or three times daily for three days, along with chlorhexidine 0.20% mouthwash twice daily for 10 days. Antibiotics were not prescribed postoperatively.

Clinical examination and suture removal were performed after 10 days. The postoperative recovery was uneventful, and no adverse reactions or complications were reported during follow-up.

After 12 weeks, CBCT examination of the areas of interest was performed prior to implant placement. A full-thickness flap was raised, and before preparation of the implant bed, a bone cylinder was obtained from each treatment group using a 2-mm trephine bur. Implant placement was then performed according to the manufacturer’s standard protocol. Finally, the flap was sutured using 4-0 polydioxanone sutures (Monosorb, Medipack, Greece). All implants received prosthetic restoration after three to four months.

### Microscopic evaluation

All bone biopsies were blindly assessed by the same senior academic oral pathologist (examiner). The examiner was calibrated (Kappa=0.91) in a pilot study relevant to the subject before the actual study.

Measurements were repeated one week after the initial examination. Microscopic evaluation was performed on the entire slide for each case by an experienced oral pathologist.

Bone biopsies were fixed in 10% buffered formalin for at least 24 hours, decalcified in a 10% EDTA-based solution, and embedded in paraffin. Five-micrometre-thick tissue sections stained with haematoxylin and eosin from each cylinder were used for the histological and histomorphometric analysis. All images were acquired using an Olympus CX23 microscope (Japan) equipped with a Basler ace acA1920-40uc digital camera (Basler, Germany) and were subsequently analyzed and digitalized. Histological assessment was performed on the entire slide and all available tissue.

The presence of the following features was assessed: new bone formation, osteoblasts, osteoclasts, osteocytes, osteolytic lacunae, and bone marrow vascularity (vascular versus non-vascular). Histological identification of these qualitative features was performed manually according to predefined histological criteria rather than by automated threshold-based image analysis. All histological sections were evaluated at an original magnification of ×100. New bone formation was assessed by histomorphometry and expressed as a percentage (%) of the total tissue area on each slide occupied by bone containing osteocyte-containing lacunae. Histomorphometric measurements were performed using the Pathomation.com, software and the quantitative data were categorized as follows: 1–25%, 26–50%, and >50%. Allograft particles, characterized by more intense basophilia and lacunae without osteocytes, were excluded from the measurements.

### Statistical analysis

Categorical histological variables were analyzed using the appropriate statistical methods, namely the chi-square test or Fisher’s exact test, when required. Fisher's exact test was applied whenever any expected cell frequency was below 5. No parametric tests were applied to categorical data. The percentage of new bone formation was recorded as a categorical variable from the outset (1–25%, 26–50%, and >50%), in accordance with Mousavi, et al.^29^ (2024), and was analyzed using categorical methods as reported by Mousavi, et al.^29^ (2024). The significance level was set at p-value 0.05.

Statistical analysis was performed using SPSS software (version 29.0, SPSS Statistics, IBM).

## Results

Figure 1 shows the CONSORT flow diagram of the study. There were no dropouts; therefore, histological evaluation was performed for all 26 specimens.

**Figure 1:**
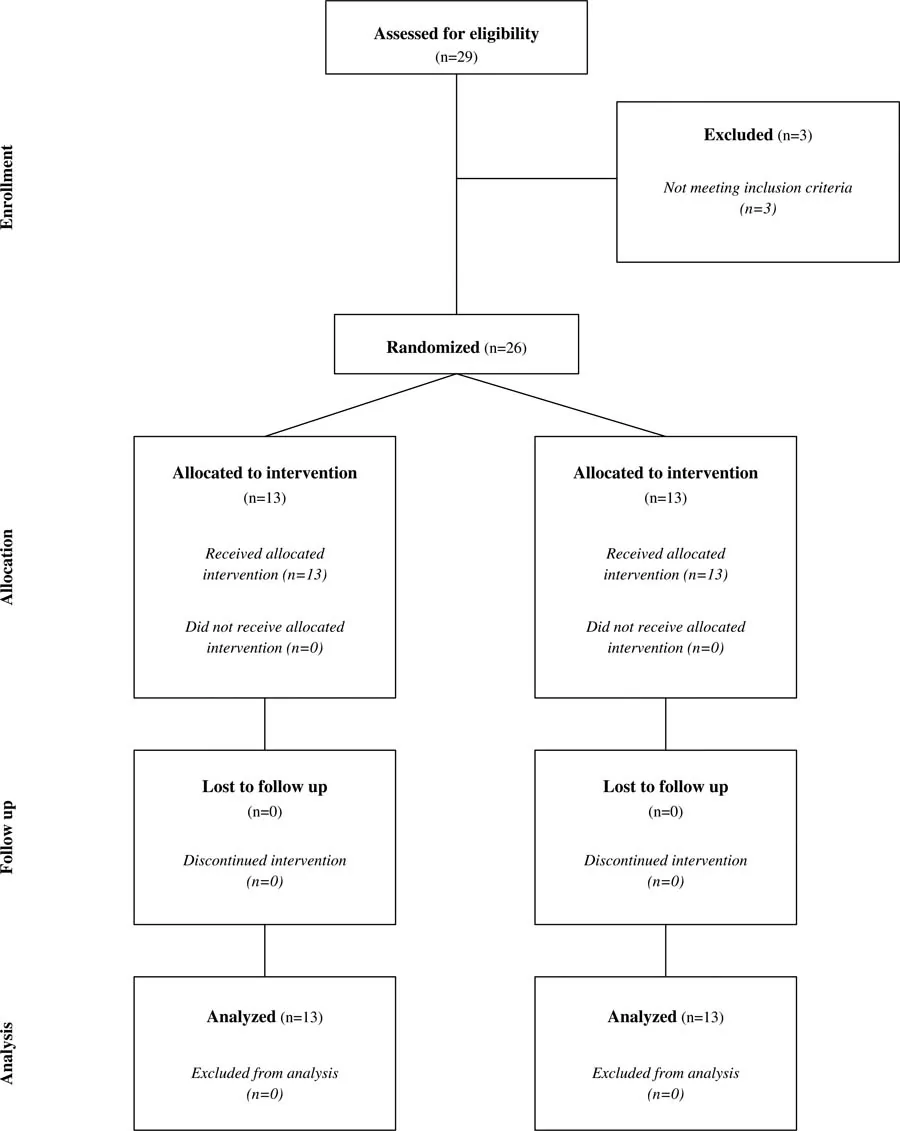
CONSORT flow diagram of the study.

For analysis, the percentage of new bone formation was divided into three subgroups: 1–25%, 26–50%, >50%. As shown in Table 1, in the experimental group, 3/13 cases (23.1%) showed 1–25% bone formation, 3/13 cases (23.1%) showed 26–50%, and 7/13 cases (53.8%) showed >50% new bone formation. In the control group, 9/13 cases (69.2%) showed 1–25% bone formation, none (0%) showed 26–50%, and 4/13 cases (30.8%) showed >50% new bone formation. The proportion of cases with >50% new bone formation was higher in the experimental group than in the control group, and this difference was statistically significant (χ^2^=6.818, p=0.033).

**Table 1:** Area of new bone formation in the control and experimental groups (Ν and %).

Treatment Group	Area of new bone formation			
	1–25%	26–50%	>50%	Total
Allograft + collagen membrane	69.2%	0%	30.8%	13
L-PRF	23.1%	23.1%	53.8%	13

Data are presented as the distribution of cases, expressed as percentages within each category of new bone formation (1–25%, 26–50%, and >50%). Statistical analysis was performed using Pearson’s chi-square test to compare groups. Statistical significance was set at p<0.05.

Figure 2 shows fibrous bone with osteocytes within bone lacunae in the experimental group after a 12-week healing period. Lamellar bone can also be observed.

**Figure 2:**
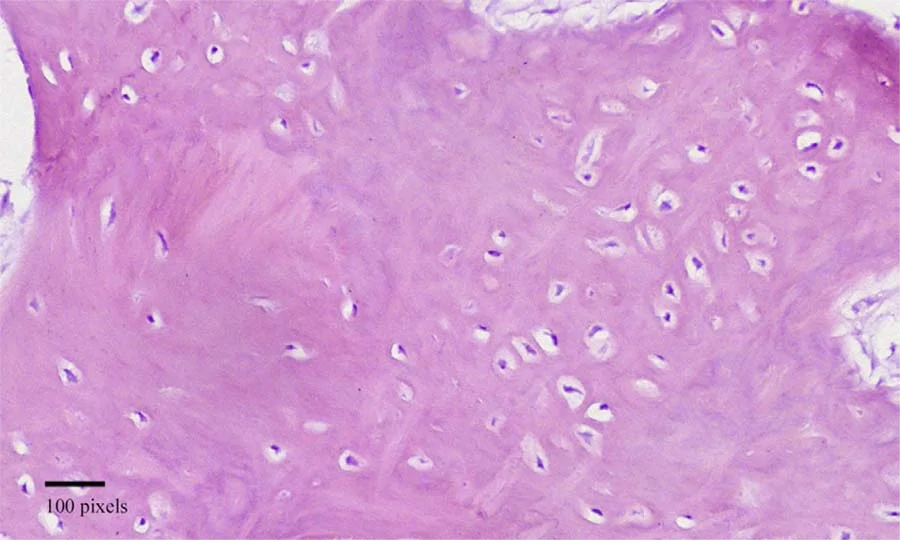
Histological findings in the experimental L-PRF group. Haematoxylin and eosin (H&E) staining. Original magnification ×100. Newly formed bone containing osteocytes within bone lacunae.

The presence of osteoblasts was detected in all cases in the experimental group (13/13, 100%), compared to 10/13 cases (76.9%) in the control group, as shown in Table 2. The difference between the experimental and control groups was not statistically significant (Fisher's exact test, p=0.220; χ^2^=3.391, p=0.066 for reference).

**Table 2:** Presence of osteoblasts in the control and experimental groups (N and %).

Treatment group	Yes (Ν/%)	No (Ν/%)	Total
Allograft + collagen membrane	10 (76.9%)	3 (23.1%)	13
L-PRF	13 (100.0%)	0 (0.0%)	13

Figure 3 presents osteoblasts (OB) lining the bone surface with increased osteoblastic activity in the experimental group.

**Figure 3:**
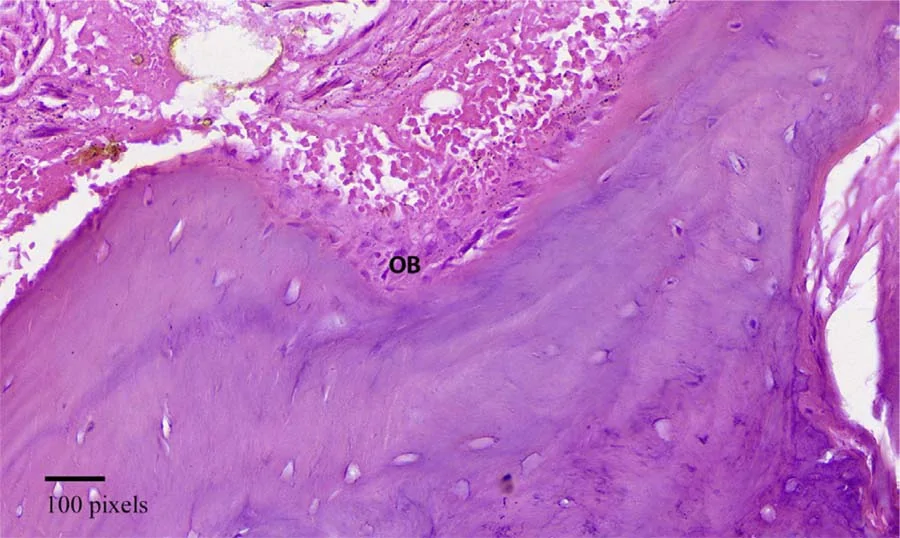
Histological findings in the experimental L-PRF group. Haematoxylin and eosin (H&E) staining. Original magnification ×100. Active osteoblasts (OB) lining the bone surface, suggestive of increased osteoblastic activity.

Table 3 shows the presence of osteoclasts. In the experimental group, osteoclasts were present in most cases (12/13 cases; 92.3%), compared to 5/13 cases (38.5%) in the control group. This difference was statistically significant (χ^2^=8.327, p=0.004). Figure 4 shows the presence of both osteoblasts and osteoclasts along the bone surface in the experimental group.

**Table 3:** Presence of osteoclasts in the control and experimental groups (N and %).

Treatment Group	Yes (N/%)	No (N/%)	Total
Allograft + collagen membrane	5 (38.5%)	8 (61.5%)	13
L-PRF	12 (92.3%)	1 (7.7%)	13

**Figure 4:**
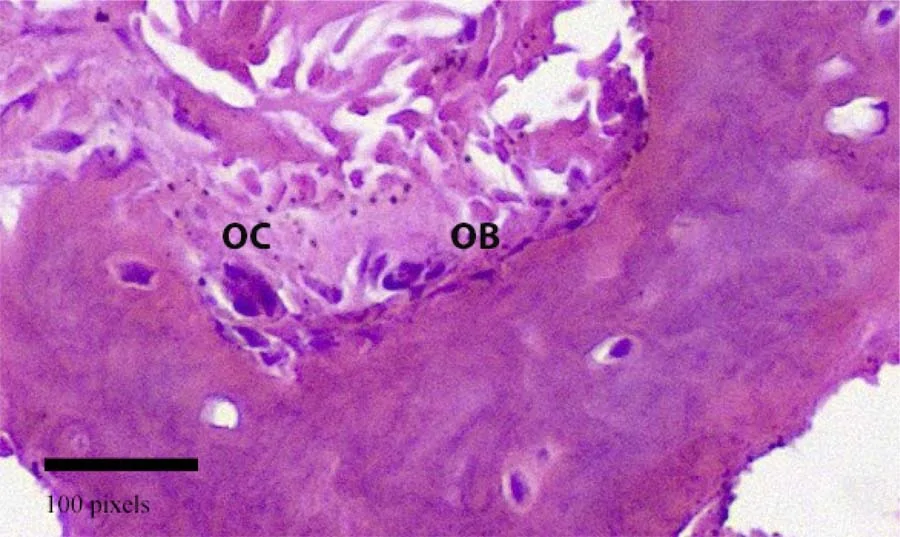
Histological findings in the experimental L-PRF group. (Haematoxylin and eosin (H&E) staining. Original magnification ×100. Simultaneous presence of osteoblasts (OB) and osteoclasts (OC) along the bone surface, indicating ongoing bone remodelling.

As shown in Table 4, osteolytic lacunae were found in the majority of cases in the experimental group, (12/13 cases; 92.3%) compared to 8/13 cases (61.5%) in the control group. This difference was not statistically significant, (χ^2^=3.467, p=0.063). The presence of osteolytic lacunae, indicative of active bone resorption in the experimental group, is shown in Figure 5.

**Table 4:** Presence of osteolytic lacunae in the control and experimental groups (N and %).

Treatment Group	Yes (N/%)	No (N/%)	Total
Allograft + Collagen Membrane	8 (61.5%)	5 (38.5%)	13
L-PRF	12 (92.3%)	1 (7.7%)	13

**Figure 5:**
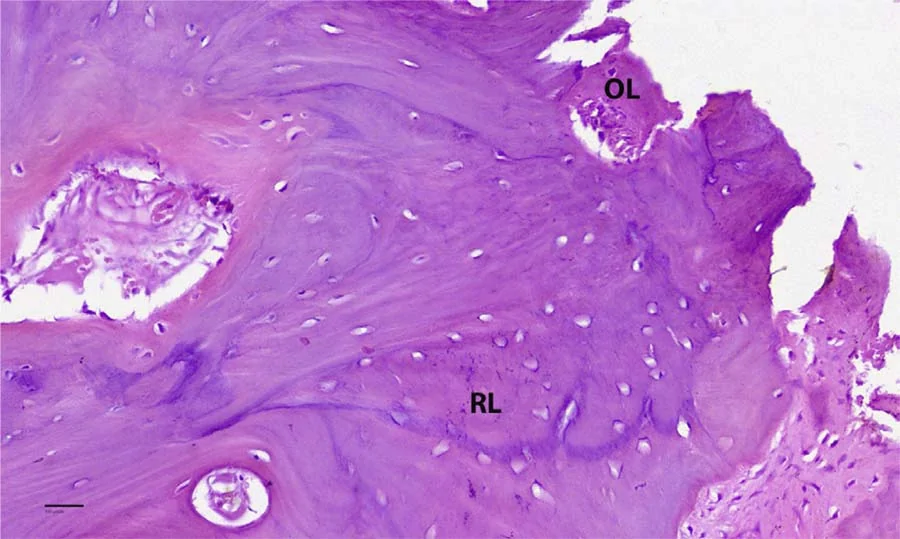
Histological section from the experimental L-PRF group demonstrating an osteolytic lacuna (OL), indicative of osteoclastic bone resorption, and reversal lines (RL), reflecting ongoing bone remodelling. Haematoxylin and eosin (H&E) staining. Original magnification ×100.

Following these findings, a statistically significant association was observed between the presence of osteoclasts and osteolytic lacunae. Additional statistical analysis was therefore performed to further assess the strength of this association (Tables 5 and 6).

To investigate the association between the presence of osteoclasts and osteolytic lacunae, a 2×2 contingency table analysis was performed, as shown in Table 5. Both variables were dichotomous categorical variables (yes/no), and the association was evaluated using Fisher's exact test, given that two of the four expected cell frequencies were below 5.

**Table 5:** Raw data on the presence of osteoclasts and osteolytic lacunae and statistical evaluation of their correlation.

Osteoclasts	Presence of Osteolytic Lacunae (N/%)	No Osteolytic Lacunae (N/%)	Total
Yes	16 (94.1%)	1 (5.9%)	17
No	4 (44.4%)	5 (55.6%)	9
Total	20 (61.5%)	6 (38.5%)	26

Fisher’s exact test (p=0.0097)

Two of the four expected cell frequencies in this table were below 5 (3.92 and 2.08); therefore, the assumptions for Pearson’s χ^2^ test were not met, and Fisher’s exact test (p=0.0097) was used as the primary test of association. Pearson’s χ^2^ is reported for reference only (χ^2^=8.180, df=1, p=0.004).

To assess the strength of the association, the Phi coefficient and Cramer’s V were also calculated (Table 6).

**Table 6:** Raw data on the presence of osteoclasts and osteolytic lacunae and statistical evaluation of their association using the Phi coefficient and Cramer’s V.

Osteoclasts	Presence of Osteolytic Lacunae (N/%)	No Osteolytic Lacunae (N/%)	Total
Yes	16 (94.1%)	1 (5.9%)	17
No	4 (44.4%)	5 (55.6%)	9
Total	20 (61.5%)	6 (38.5%)	26

Phi = 0.561, p=0.0097 (Fisher’s exact) Cramer’s V = 0.561, p=0.0097 (Fisher’s exact)

Note: as Phi and Cramer’s V for a 2×2 table are mathematically derived from the χ^2^ statistic, their associated significance level corresponds to the same test used in Table 5.

As shown in Table 7, osteocytes were detected in all cases in the experimental group (13/13, 100%), compared to 9/13 cases (69.2%) in the control group. Given expected cell frequencies below 5 in this table, Fisher's exact test was used; this difference did not reach statistical significance (p=0.096; χ^2^=4.727, p=0.03 for reference).

**Table 7:** Presence of osteocytes in the control and experimental groups (N and %).

Treatment Group	Yes (N/%)	No (N/%)	Total
Allograft + collagen membrane	9 (69.2%)	4 (30.8%)	13
L-PRF	13 (100.0%)	0 (0.0%)	13

Lamellar bone with discrete reversal lines and osteocytes within bone lacunae were observed in the experimental group. Figure 6 shows lamellar bone with discrete reversal lines (RL) and allograft particles (AG) in the control group. The allograft particles remained clearly identifiable after a 12-week healing period.

**Figure 6:**
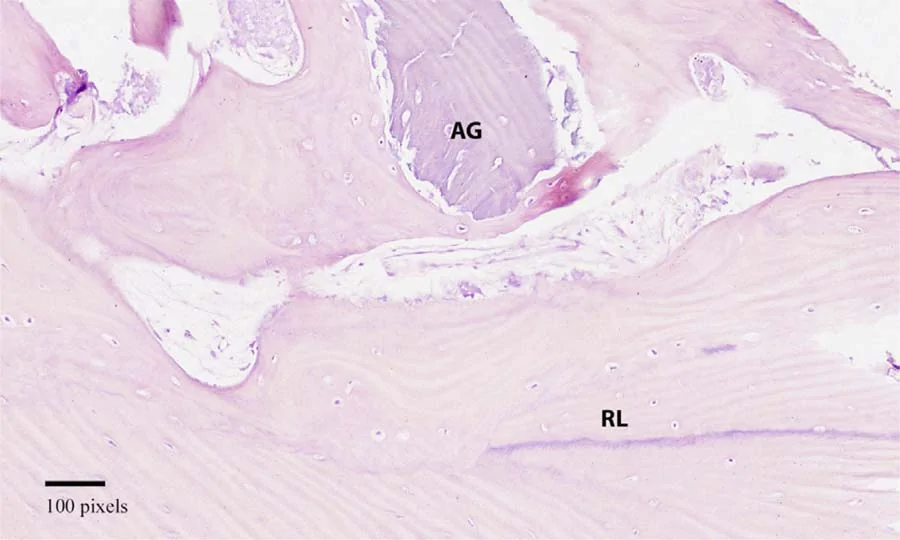
Histological section from the control group showing lamellar bone with osteocytes embedded within bone lacunae, discrete reversal lines (RL), and residual allograft particles (AG). Haematoxylin and eosin (H&E) staining. Original magnification ×100.

Finally, as shown in Table 8, the bone marrow was classified as vascular in the majority of cases in the experimental group (11/13, 84.6%), compared to 5/13 cases (38.5%) in the control group (χ^2^=5.85, p=0.016). The increased vascularity in the experimental group, characterized by newly formed blood vessels (BV), is shown in Figure 7.

**Table 8:** Presence of blood vessels in the control and experimental groups (N and %).

Treatment Group	Yes (N/%)	No (N/%)	Total
Allograft + collagen membrane	5 (38.5%)	8 (61.5%)	13
L-PRF	11 (84.6%)	2 (15.4%)	13

**Figure 7:**
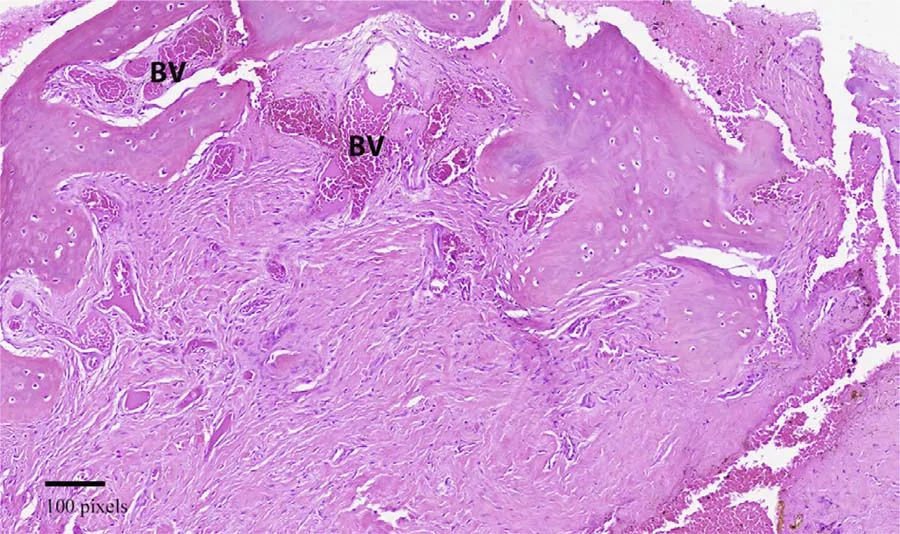
Histological section from the experimental L-PRF group demonstrating newly formed blood vessels (BV) within the connective tissue and adjacent newly formed bone, indicative of enhanced vascularisation and angiogenic activity during socket healing. Haematoxylin and eosin (H&E) staining. Original magnification ×100.

## Discussion

In this RCT, the histological features of post-extraction sockets following L-PRF application were compared with those observed following the use of an allograft and collagen membrane, as the latter is considered a widely accepted and valuable technique for ARP.^3,8,10,11,30,31^

Systematic reviews have confirmed that ARP significantly reduces ridge dimensional loss compared to natural healing.^10,32^ Numerous randomized controlled clinical trials and meta-analyses have evaluated the effects of ARP using allografts on ridge dimensions. A landmark systematic review by Avila-Ortiz, et al.^10^ (2014) found that socket grafting procedures with allograft materials has a significant treatment effect in buccolingual width (1.89mm), midlingual height (1.18mm) and midbuccal height (2.07mm) compared to spontaneous healing. Histological studies assessing sockets grafted with FDBA have demonstrated substantial new bone formation, with close contact between newly formed bone and residual graft particles, demonstrating favorable osseointegration.^11,31^ Iasella, et al.^8^ (2003), in a comparative clinical and histological study in humans evaluating alveolar ridge preservation using allograft and collagen membrane versus natural healing, assessed bone vitality and the presence of osteoblasts in both groups.^8^ Histological analysis revealed a greater amount of bone in the alveolar ridge preservation group, with 28% vital bone and 37% non-vital FDBA fragments.^8^ Piattelli, et al.^33^ (1996) evaluated DFDBA and FDBA in post-extraction sockets and concluded that, in bone biopsies from FDBA-treated sites, many particles were surrounded by newly formed bone.^33^

The use of platelet concentrates produced without anticoagulant agents for ARP to improve bone healing has gained increasing interest in recent years.^25,34,35^ Specifically, regarding L-PRF in post-extraction sockets, Serafini, et al.^36^ (2020) reported that post-extraction sockets filled with L-PRF presented mature trabecular bone with organized marrow spaces and no residual fibrin three months after extraction, indicating complete resorption of the material and biocompatibility. Canellas et al.^28^ (2020), in an RCT, found that post-extraction sockets filled with L-PRF showed considerably higher percentages of newly formed bone and less connective tissue than those undergoing natural healing, although radiographic ridge dimensions did not differ significantly between groups.

To the authors' knowledge, only one previous clinical study has performed a histomorphometric comparison of post-extraction sockets treated with PRF versus an allograft combined with a collagen membrane.^37^Ivanova, et al.^37^(2021) reported a significant difference in the amount of newly formed bone between the control group and both treatment groups (PRF and allograft). In this study, L-PRF-treated extraction sockets showed a greater vital bone surface area than sockets treated with an allograft and collagen membrane, and this difference was statistically significant. The present results are also in accordance with the findings of Castro et al.^23^, (2021), who reported the application of L-PRF in extraction sockets resulted in almost 50% more new bone formation compared to natural healing. Similarly, Mousavi, et al.^29^ (2024) showed that new bone formation exceeded 50% in 25% of the L-PRF samples, whereas none of the samples in the natural healing group exceeded 50%. The enhanced osteogenesis observed following L-PRF application may be associated with the osteogenic potential of platelet derivatives rich in growth factors such as PDGF, VEGF, and TGF-β1, which may contribute to osteoblast recruitment.^26,29,38^ Castro, et al.^23^ (2021) demonstrated that the growth factors present in L-PRF may accelerate bone remodelling and osseointegration. Consistent with the proposed osteogenic potential of L-PRF, this study detected osteoblasts in 100% of L-PRF-treated sockets, compared to 76.9% of sockets filled with an allograft and covered with a collagen membrane. Overall, it may be hypothesized that L-PRF promotes osteogenesis by means of osteoblast formation, potentially contributing to faster bone healing. However, this hypothesis warrants confirmation in future studies.

Park et al.^39^ (2022) conducted a preclinical animal study to histomorphometrically assess the effects of L-PRF alone or combined with a grafting material in post-extraction sockets in canine models. L-PRF-treated sockets yielded a higher level of parallel-aligned collagen fibers, fewer osteoblasts, and the presence of cutting cones produced by osteoclasts, indicating bone remodelling, compared to grafted sockets in the control group.^39^ In accordance with these findings, this study revealed that a higher percentage of post-extraction sockets treated with L-PRF showed osteoclasts and osteolytic lacunae around newly formed vital bone, indicating greater osteoclastic activity. It is acknowledged that increased numbers of osteoclasts and the presence of osteolytic lacunae may reflect active bone turnover and ongoing remodelling, which may be associated with accelerated healing.^40^ However, these findings may also indicate a less mature stage of bone remodelling rather than fully mature bone architecture.^23,25,41^

The bone marrow of L-PRF-treated sockets showed greater vascularity compared to the control group, which is in accordance with previous studies.^25,34^

The main strength of this study is that it reported qualitative histological parameters in L-PRF-treated post-extraction sockets compared to sockets treated with an allograft and collagen membrane. The sample size was determined through power analysis to detect potential statistically significant differences. Additionally, several histological features assessed in this study, including the presence of osteoclasts, osteocytes, osteolytic lacunae, and blood vessels, have not been evaluated in previous clinical studies. However, clinical studies regarding ridge preservation techniques are complex because factors related to socket anatomy, such as bone thickness, fenestrations and dehiscence, periodontal biotype, and location (maxilla or mandible), may affect the results. An ideal study design would have included a split-mouth design with identical extraction sockets. However, in clinical practice, it is extremely difficult to find patients requiring extraction of teeth with comparable socket anatomy and similar residual alveolar bone surrounded by healthy adjacent teeth.

A potential limitation of this study is the use of non-parametrical statistical methods (chi-square or Fisher’s exact test, as appropriate) to assess categorical histological variables. The percentage of new bone formation was recorded from the outset as a categorical variable (1–25%, 26–50%, and >50%), according to the predefined histological assessment protocol.^29^

The sample size calculation was based on a large standardized effect size (Cohen’s d = 0.80), informed by the favorable outcomes reported in previous L-PRF trials, rather than by quantitative effect-size data (means and standard deviations) extracted directly from those studies. Therefore, this assumption may have been optimistic.

Additionally, the categorical scale used to analyze the primary outcome does not fully align with the continuous mean-difference framework on which the original power calculation was based. Together, these considerations suggest the study may lack sufficient power to detect smaller yet clinically meaningful differences between groups, and that the reported statistical comparisons should therefore be interpreted cautiously. These findings are best regarded as hypothesis-generating, offering preliminary estimates of effect size and variability to inform the design of future, adequately powered trials using consistent outcome measures.

The results suggest that ARP with L-PRF may be associated with accelerated healing, improved bone quality, and a higher rate of bone remodelling. These findings reflect the investigative and exploratory nature of the study. The histological findings support the need for additional studies, preferably with larger sample sizes. Moreover, future studies incorporating radiographic evaluation alongside clinical and histological assessments could further corroborate these observations.

## Conclusions

Within the limitations of this randomized controlled trial comparing L-PRF with an allograft combined with a collagen membrane for ARP in post-extraction sockets, the following conclusions can be drawn:

- The percentage of new bone formation was significantly higher in the experimental group than the control group.
- The presence of osteoclasts, osteocytes, and blood vessels was significantly higher in the experimental group than in the control group.
- There was no statistically significant difference in the presence of osteoblasts, osteocytes and osteolytic lacunae between the experimental and control groups. However, given that the sample size was calculated to detect a large effect for the primary outcome, this study may have been underpowered to identify smaller but potentially relevant differences in these secondary parameters. Therefore, these findings should not be interpreted as evidence of true equivalence.
- A significant correlation between the presence of osteoclasts and osteolytic lacunae was observed in all cases, a finding that has not been investigated in previous studies.
- Overall, evaluation of the histological parameters investigated suggests a potentially positive contribution of L-PRF to bone healing. Given the exploratory nature of the effect-size assumptions underlying this study design, these results should be considered preliminary and warrant confirmation in future, adequately powered trials designed to detect more modest effect sizes.
